# Can delivery systems use cost-effectiveness analysis to reduce healthcare costs and improve value?

**DOI:** 10.12688/f1000research.7531.1

**Published:** 2016-10-25

**Authors:** Lucy A. Savitz, Samuel T. Savitz

**Affiliations:** 1 Institute for Healthcare Delivery Research, Intermountain Healthcare, Salt Lake City, Utah, USA; 2 Department of Health Policy and Management, School of Public Health, University of North Carolina at Chapel Hill, Chapel Hill, North Carolina, USA

**Keywords:** Healthcare cost, cost-effectiveness, delivery systems, Cost-effectiveness analysis, health outcomes

## Abstract

Understanding costs and ensuring that we demonstrate value in healthcare is a foundational presumption as we transform the way we deliver and pay for healthcare in the U.S. With a focus on population health and payment reforms underway, there is increased pressure to examine cost-effectiveness in healthcare delivery. Cost-effectiveness analysis (CEA) is a type of economic analysis comparing the costs and effects (i.e. health outcomes) of two or more treatment options. The result is expressed as a ratio where the denominator is the gain in health from a measure (e.g. years of life or quality-adjusted years of life) and the numerator is the incremental cost associated with that health gain. For higher cost interventions, the lower the ratio of costs to effects, the higher the value. While CEA is not new, the approach continues to be refined with enhanced statistical techniques and standardized methods. This article describes the CEA approach and also contrasts it to optional approaches, in order for readers to fully appreciate caveats and concerns. CEA as an economic evaluation tool can be easily misused owing to inappropriate assumptions, over reliance, and misapplication. Twelve issues to be considered in using CEA results to drive healthcare delivery decision-making are summarized. Appropriately recognizing both the strengths and the limitations of CEA is necessary for informed resource allocation in achieving the maximum value for healthcare services provided.

## Rationale for cost-effectiveness

Understanding costs and ensuring that we demonstrate value in healthcare is a foundational presumption as we transform the way we deliver and pay for healthcare in the U.S. The consideration of cost as a primary research outcome in comparative effectiveness research was all but banned in the Affordable Care Act; this coupled with the fact that the Food and Drug Administration approvals and Medicare payment decisions do not consider cost has been pointed to as a key factor in rising U.S. healthcare costs. With a focus on population health and payment reforms underway, there is increased pressure to examine cost-effectiveness in healthcare delivery.

## Background

Cost-effectiveness analysis (CEA) is a type of economic analysis comparing the costs and effects (health outcomes) of two or more treatment options. The result is expressed as the incremental cost-effectiveness ratio, where the denominator is the incremental gain in health from a measure (e.g. years of life or quality-adjusted years of life) and the numerator is the incremental cost associated with that health gain. For higher cost interventions, the lower the ratio of costs to effects, the higher the value.

Research on the value of healthcare services helps to direct finite resources towards their most efficient use. The common economic research approaches (see Table 1) differ in how they combine information on costs and outcomes. Traditional comparative effectiveness research and outcomes research focus on health outcomes or resource utilization in non-monetary units. Cost minimization compares alternatives that have roughly similar outcomes and identifies the approach that minimizes costs. Cost-benefit analysis incorporates outcomes by converting them into monetary terms. This research can be challenging, since many health outcomes do not have an obvious monetary amount associated with them, although values based on willingness to pay for health risk reductions are often used to monetize health gains. CEA measures the ratio of incremental costs to health outcomes such as life years. While cost-utility analysis also computes a ratio, the health outcomes are specifically measured in terms that reflect both survival and quality of life. Research in developed countries typically uses quality-adjusted life years (QALYs) as the utility measure. QALYs weight life years by utility weights, which typically range from zero to one, with zero representing death and one representing perfect health. Even though cost-utility analysis is a special type of CEA, many researchers refer to both methods as CEA ^1^.

**Table 1.** Methods for calculating value in healthcare.

Method	Costs	Outcomes	Example Measures
Comparative effectiveness research & outcomes research	N/A	Health outcomes or utilization	Life years Disease incidence Quality of life Hospitalizations
Cost minimization	$	N/A	$
Cost benefit	$	$	$
Cost effectiveness	$	Health outcomes or utilization measures	$/life years $/hospitalization
Cost utility	$	Quality-adjusted life years or disability-adjusted life years	$/quality-adjusted life year $/disability-adjusted life year

Source: Adapted from Drummond, 2015

Important considerations for cost-effectiveness studies include the perspective, time horizon, population, and alternatives ^1, 2^. The perspective refers to the entity for whom costs and benefits accrue. The societal perspective is sometimes presented as the ideal ^1^. However, it is difficult to measure all conceivable costs and benefits for society, and no single decision-maker is charged with optimizing societal welfare. Common alternative choices for perspective include a healthcare delivery system, payers, and government agencies (e.g. the Centers for Medicare & Medicaid Services). The perspective determines which costs and benefits are relevant to include. For example, the societal perspective incorporates loss to productivity for patients and caregivers that would not be fully captured by other perspectives. Also, the payer perspective incorporates the reimbursed or contractual amount to be paid even though that amount is usually different to the actual costs of treatment to the delivery system ^1^. The time horizon is important because it determines how far into the future costs and outcomes are included. Future costs and outcomes are typically discounted at 3 percent to reflect societal preferences for present benefits relative to future benefits ^3^. The population should represent those patients who are eligible to receive the treatment. Therefore, one must specify clinical factors such as age, disease severity, and co-morbidities that make patients eligible for receipt of the treatment ^1^, and Accountable Care Organizations (ACOs) are responsible for only an assigned population living in a limited geographic area. Clear specification of the patient population lets readers know to whom the analysis applies. Treatment alternatives must also be clearly specified. In general, cost-effectiveness research compares new therapies, diagnostic procedures, or preventive services to usual care. It is typically inappropriate to compare a new therapy to no treatment as patients almost always receive some type of care ^1, 2^.

Cost-effectiveness research typically uses data from a single trial or data from multiple studies reported in the literature. In some cases, researchers with clinical trial data will evaluate the cost-effectiveness using the costs and benefits from each treatment arm, although such a CEA would apply only to the population and conditions studied in the trial. In order to reflect a broader evidence base that includes multiple trials, observational data, and outcomes that apply to different populations and to time horizons beyond those of individual studies, researchers have developed simulations models. These models attempt to capture the key disease states and clinical strategies. They will then assign parameter values for transition probabilities, costs, and utilities. Common types of simulation models include decision trees and Markov models. Decision models allow researchers to combine data from across the literature into a single analysis. It also allows researchers to incorporate uncertainty with regard to parameter values in sensitivity analyses ^1, 2^.

The primary outcome of cost-effectiveness research is the incremental cost-effectiveness ratio (ICER). The ICER is calculated as follows:

$$\frac{{\text{Cost}}_{\text{Novel}}–{\text{Cost}}_{\text{Usual}\;\text{Care}}}{{\text{Effect}}_{\text{Novel}}–{\text{Effect}}_{\text{Usual}\;\text{Care}}}$$

The calculation becomes more complex when multiple interventions are being compared (for more information, see 1).

When costs are measured in dollars and effects in QALYs, the ICER is measured as $/QALY. Researchers commonly compare the ICER to thresholds to facilitate comparisons. Common thresholds include $50,000/QALY, $100,000/QALY, and $150,000/QALY ^4^. These thresholds are arbitrary and similar to the 0.05 p-value cut-off for statistical significance. However, like p-values, the thresholds can be useful as a way to communicate results. Some researchers have attempted to make thresholds more reasoned by basing them on the cost-effectiveness ratios for programs that are currently funded ^5^.

**Figure 1.**
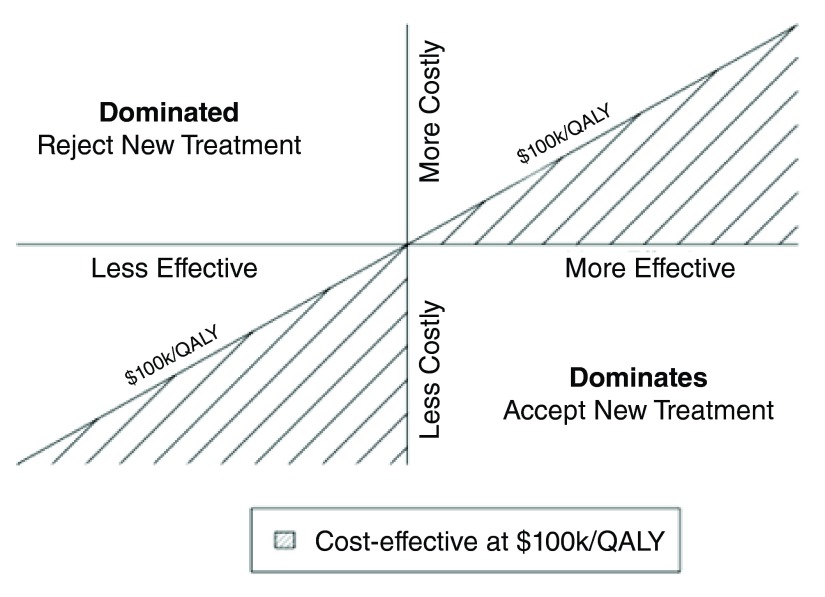
Cost-effectiveness plane. QALY; quality-adjusted life year.

A common way to communicate the results is by placing them on the cost-effectiveness plane ( Figure 1). The plane has four quadrants that represent the possible results:

1) Upper right: more costly/more effective
2) Upper left: more costly/less effective
3) Lower left: less costly/less effective
4) Lower right: less costly/more effective

Dominance occurs in the upper left and lower right quadrants. This result means that the new treatment is more costly and less effective than usual care (upper left) or the new treatment is less costly and more effective than usual care (lower right). Therefore, the preferred alternative is unambiguous. Ambiguity exists in the upper right and lower left quadrants. The preferred alternative depends on the tradeoff between health outcomes and costs. In the diagram, the line represents a threshold of $100,000/QALY. This threshold specifies a particular tradeoff for costs and outcomes. Using this threshold, treatments would be cost-effective if their ICERs were below $100,000/QALY and they were in the upper right quadrant. Treatments would also be cost-effective if the ICER was greater than $100,000/QALY and the treatment was in the lower left quadrant.

CEA has the potential to identify clinical strategies that improve health while maintaining or even reducing costs. Adopting interventions in the lower right quadrant or eliminating interventions in the upper left quadrant of Figure 1 would both reduce costs and improve health. Alternatively, CEA can be used to prioritize resources by reallocating from interventions with higher ICERs to interventions with lower ICERs. The reallocation of resources in this manner would also result in reduced costs and improved health outcomes ^6^.

## Caveats and concerns to consider

While CEA is not new, the approach continues to be refined with enhanced statistical techniques and standardized methods ^1^. A set of companion articles published in *Circulation: Cardiovascular Quality and Outcomes* examines the pros and cons of CEA ^7, 8^. Some of the valid concerns and cautions described in these papers are summarized below:

1. Valid measures of both effectiveness and cost are subject to considerable variation in methods used
2. The time horizon needed often extends beyond the timeframe for which data are available
3. Studies based only on the results of trials often suffer from potential bias and limited generalizability
4. Modeling and simulation-based approaches are only as good as the input data, which are often extremely limited in areas of innovation such as treatment techniques, pharmaceuticals, diagnostics, and devices
5. Appropriateness of the status quo as the comparator relies on an assumption that standard or usual care is at least somewhat cost-effective
6. Because inputs to CEA are associated with uncertainty, the results of a CEA should also reflect this uncertainty using available methods
7. There is no generally accepted ICER threshold in the U.S.
8. When quality of life is reflected in the QALY measure, then all life years in the CEA should be adjusted for quality to avoid mixing of unadjusted life years and QALYs
9. CEA based on life years or QALYs values a year of a person’s life equally across all ages, diseases, or medical interventions
10. CEA studies that use disease-specific outcome measures in the denominator conducted cannot be meaningfully compared across varied clinical areas
11. CEA studies that use simulation studies often make critical assumptions about the disease process, costs incurred, and quality of life; the assumptions should be plausible given what is known
12. CEA studies should be transparent about the key assumptions that are made and how those assumptions may affect the results

CEA as an economic evaluation tool can be easily misused owing to inappropriate assumptions, over reliance, and misapplication. There is a tendency to assess the cost to treat as an accounting exercise, but identification of relevant costs should consider economic theory and the perspective of the impacted entity. For a healthcare delivery system relying on CEA, decisions are often made based on results of studies using a societal or payer perspective. The structure of delivery systems matters and institutional modifications or new indications are not typically accounted for in CEA. Nevertheless, we should regard CEA as a useful tool that can inform the allocation of resources in a delivery system setting.

## Conclusions

CEA is one of many considerations when allocating healthcare resources, as Hadorn recognized from the Oregon coverage ranking experience in the early 1990s ^9^, and not one to solely rely upon to reconfigure the healthcare delivery system. CEA is meaningfully applied on a case-by-case basis and, at that level, can be used to inform value-based decision-making. Most recently, it has been used by commercial insurers and self-funded employer-based plans to make determinations on coverage decisions for high-cost drugs such as hepatitis C treatment ^10^. As a tool to reduce costs more globally to delivery systems, CEA is unlikely to singularly serve in that role, as there are an infinite number of treatments to assess, and one can easily envision exceeding a budget based on all of those interventions that pass a subjective criterion. Appropriately recognizing both the strengths and the limitations of CEA is necessary for informed decision-making in achieving the maximum value for healthcare services provided.
